# Platelet transfusion in end-of-life adult care

**DOI:** 10.1016/j.htct.2025.106228

**Published:** 2025-12-17

**Authors:** Diana Cibele, Fernanda Trigo, Miguel Barbosa, Jorge Almeida, José Artur Paiva, Edna Gonçalves, João Brito, José Teixeira, Fernando Araújo

**Affiliations:** a Transfusion Medicine and Blood Bank, Center of Thrombosis and Hemostasis, São João Local Health Unit, Porto, Portugal; b Hematology Department, São João Local Health Unit, Porto, Portugal; c Oncology Department, São João Local Health Unit, Porto, Portugal; d Internal Medicine Department, São João Local Health Unit, Porto, Portugal; e Faculty of Medicine - University of Porto, Portugal; f Intensive Care Medicine Department, São João Local Health Unit, Porto, Portugal; g Palliative Care Deparment, São João Local Health Unit, Porto, Portugal; h Humanization Department, São João Local Health Unit, Porto, Portugal; i Religious and Spiritual Assistance Department, São João Local Health Unit, Porto, Portugal

Dear Editor,

In a tertiary care hospital, where the complexity of patients treated has increased over the years, the decision to transfuse each individual that presents with anemia or thrombocytopenia must be guided not only by clinical and laboratory characteristics, but also by the availability of blood products, ensuring judicious and ethical use of this scarce resource. In our hospital, despite a continuous effort to increase platelet collections, transfusion demands continue to rise, requiring careful and continuous reassessment of transfusion practices.

Current protocols recommend platelet transfusion in case of active hemorrhage or, prophylactally, for high-risk bleeding procedures in patients with severe thrombocytopenia or under antiplatelet therapy, and for spontaneous bleeding prevention in severe thrombocytopenia cases [1, 2, 3]. In asymptomatic patients with platelet counts <10 × 10^3^/µL or <20 × 10^3^/µL if other bleeding risk factors exist, platelet transfusion is intended, in particular, to reduce the likelihood of intracerebral bleeding [1, 2, 3, 4].

However, emerging evidence suggests that transfusion policies should not rely solely on platelet counts, but instead be adapted to each patient’s bleeding risk (also associated with personal history of bleeding, renal failure, hypoalbuminemia, fever, or recent stem cell transplantation [5]) and care objectives, particularly in palliative and end-of-life settings [6,7].

In this population, prophylactic transfusions raise significant clinical and ethical concerns: based on the stage of the disease and estimated life expectancy, the level of care should focus on optimizing quality of life. In these cases, efforts should be made to discontinue all treatments that do not directly contribute to the patient's comfort and that do not aim for symptom control or achieving realistic goals [8,9].

In hemato-oncologic terminal patients, disease-specific treatments are often continued, resulting in frequent hospital visits and admissions, intensive care interventions, and high intrahospital mortality rates [10,11]. Unlike erythrocyte transfusions, that may provide symptomatic relief, prophylactic platelet transfusion has not consistently been associated with a reduction in bleeding complications or an improved survival in terminal patients [5,12,13].

It is known that terminal patients often face repeated hospital visits for transfusions, resulting in discomfort and stress for them and their caregivers. Nonetheless, each transfusion carries approximately a 1% risk of severe adverse reactions, and its hemostatic effect is short-lived [14, 15, 16, 17, 18]. As such, providing platelet transfusions in end-of-life care involves complex ethical principles:

• **Non-maleficence:** Restrictive prophylactic transfusion policies may prevent harm by avoiding frequent hospital trips, invasive testing, and treatment-related adverse effects that offer limited benefit in late-stage disease. Unnecessary transfusions may delay timely transition to palliative care, potentially diminishing quality of life.
• **Beneficence:** Avoiding futile treatments helps spare patients from side effects and unnecessary interventions, promoting well-being.
• **Justice:** Platelet components are scarce; clinical judgment should ensure equitable distribution while addressing individual needs.
• **Autonomy:** Patients must receive complete information about transfusion risks and benefits, allowing their preferences and advance directives to guide treatment decisions.

Based on a literature review and multidisciplinary discussion at our hospital, we proposed the following recommendations for platelet transfusion in adult patients in end-of-life care:

**Recommendation 1:** Clinical records must identify their disease stage and care objectives (curative intent, symptom control, or comfort measures only). Before deciding to transfuse platelet components, information concerning patients’ indication for advanced life support and/or prognosis-modifying therapy is essential.

**Recommendation 2:** The decision to transfuse platelet components should focus on patients’ comfort, burden of associated symptoms, and life goals.

**Recommendation 3:** Prophylactic platelet transfusions should be avoided:

- Decision should not depend on the patient’s platelet count.
- In this context, peripheral blood smear platelet counts should not be performed.

**Recommendation 4:** In patients that present with bleeding symptoms or need to undergo high bleeding risk procedures:

- Clinical justification for invasive procedures with associated bleeding risk must be weighed against their potential futility, inherent risks, and associated discomfort for the patient [13]. All measures that represent therapeutic obstinacy should be avoided.
- Patient and platelet components should not be phenotyped/genotyped for platelet and HLA antigens/genetics.
- Platelet function tests should not be performed.
- Transfusion of platelet pools should be preferred, avoiding the use of single platelet concentrates.

**Recommendation 5:**

- Each patient’s autonomy must be respected, ensuring the right to information and active participation in decisions regarding platelet transfusions, through truly informed consent.
- Considering each patient’s autonomy, advance directives, when available, must be taken into consideration in situations where the patient is unable to express his wishes.

In conclusion, we believe that the management of platelet transfusion in end-of-life care requires a nuanced, ethically grounded approach that balances clinical benefit with the patient’s comfort, dignity, and autonomy [19]. These recommendations aim to guide clinicians in judiciously using platelet components, avoiding non-symptom-relieving interventions, and promoting individualized, compassionate care for terminally ill adult patients.

## Financial disclosure and funding

None to declare.

## Informed consent

Not applicable.

## Author contributions

DC reviewed the literature and drafted the manuscript; FT, MB, JA, JAP, EG, JB and JT contributed to the data discussion; FA conceptualized the study, evaluated the data, and revised the manuscript.

## Data availability

The authors declare that data supporting the findings of this study are available within the article.

This study was approved by São João Local Health Unit Health Ethics Committee.

## Conflicts of interest

None to declare.
